# LINKS BETWEEN SELF-REPORTED HEARING WITH LEARNING AND MEMORY IN THE HEALTH AND RETIREMENT STUDY

**DOI:** 10.1093/geroni/igae098.3049

**Published:** 2024-12-31

**Authors:** Ariana Stickel, Carlos Araujo-Menendez, Carmen Chek, Laura Coco, Hector González, Wassim Tarraf

**Affiliations:** San Diego State University, San Diego, California, United States; SDSU/UC San Diego, San Diego, California, United States; Wayne State University, Detroit, Michigan, United States; San Diego State University, San Diego, California, United States; University of California San Diego, La Jolla, California, United States; Wayne State University, Detroit, Michigan, United States

## Abstract

Growing evidence links hearing loss to increased risk for dementia, but the influence of sociodemographic characteristics (e.g., race/ethnicity, insurance status) on this relationship is understudied. Therefore, we examined the relationships between self-rated hearing with learning and memory in a large, population-based sample. Participants included 3,417 individuals (mean age = 74.5 years, SD = 7.9; 9.4% Black, 8.1% Latino, 82.5% White) from the Health and Retirement Study who also completed the Harmonized Cognitive Assessment Protocol (HCAP) in 2016. Individuals rated their hearing as “excellent”, “very good”, “good”, “fair”, or “poor”. Outcomes of interest included scores from each of the learning (1-3) and the delayed memory trials from the Consortium to Establish a Registry for Alzheimer’s Disease (CERAD) Word List Memory Task. Probability weighted linear regression models were fit to examine the associations between self-reported hearing with learning and memory. We fit a series of models adjusting for hearing aid use (M1), M1 + education (M2), and M2 + race/ethnicity, age, sex, and insurance (M3). Fair and poor self-reported hearing were associated with a steeper drop from trial 3 recall to delayed recall compared to those who reported excellent hearing in fully-adjusted models (M3: Δ=-0.27, se=0.12, p < 0.05; Δ=-0.47, se=0.16, p < 0.01, respectively). Self-reported poor hearing was associated with worse delayed recall after taking into account hearing aid use and several sociodemographic factors. Hearing loss may be a ubiquitous risk factor for worse delayed recall, suggesting an increased risk for Alzheimer’s disease.

